# RETRACTION: Effect of wound infiltration of dexmedetomidine in lumbar spine surgery on postoperative wound pain: A meta‐analysis

**DOI:** 10.1111/iwj.70111

**Published:** 2024-10-16

## Abstract

Retraction: WangX. , PengY. , SiY. , and HuX. , “Effect of Wound Infiltration of Dexmedetomidine in Lumbar Spine Surgery on Postoperative Wound Pain: A Meta‐Analysis,” International Wound Journal 21, no. 3 (2024): e14523, 10.1111/iwj.14523.38050653 PMC10898393

The above article, published online on 05 December 2023, in Wiley Online Library (http://onlinelibrary.wiley.com/), has been retracted by agreement between the journal Editor in Chief, Professor Keith Harding; and John Wiley & Sons, Ltd. It came to the publisher's attention from a third party that a number of articles shared concerning similarities in format and structure. Following an investigation by the publisher, the retraction has been agreed on as the peer review and publishing process for this article were found to be manipulated. The authors did not respond to the notice of retraction.

Retraction: X. Wang , Y. Peng , Y. Si , and X. Hu , “Effect of Wound Infiltration of Dexmedetomidine in Lumbar Spine Surgery on Postoperative Wound Pain: A Meta‐Analysis,” International Wound Journal 21, no. 3 (2024): e14523, 10.1111/iwj.14523.38050653 PMC10898393

The above article, published online on 05 December 2023, in Wiley Online Library (http://onlinelibrary.wiley.com/), has been retracted by agreement between the journal Editor in Chief, Professor Keith Harding; and John Wiley & Sons, Ltd. It came to the publisher's attention from a third party that a number of articles shared concerning similarities in format and structure. Following an investigation by the publisher, the retraction has been agreed on as the peer review and publishing process for this article were found to be manipulated. The authors did not respond to the notice of retraction.

